# The Importance of the Parent–Clinician Dyad During a Neurodevelopmental Assessment in a Community Mental Health Setting

**DOI:** 10.3390/bs16101807

**Published:** 2026-10-02

**Authors:** Liza Hopkins, Michelle Kehoe, Jennifer Harrison, Mary Fleming, Richard Whitehead

**Affiliations:** 1 Infant, Child and Youth Area Mental Health and Wellbeing Service, Bayside Health, Melbourne 3189, Australia; 2 Department of Occupational Therapy, Monash University, Peninsula Campus, Frankston 3199, Australia; 3 School of Psychology, Faculty of Health, Deakin University, Burwood 3125, Australia

**Keywords:** autism, ASD, autism assessment, parent–clinician dyad

## Abstract

Although much is known about the challenges clinicians and parents face during a neurodevelopmental assessment, to date, there has been little focus on examining these differing experiences. The aim of this study was to explore the views of clinical staff and parents during an autism spectrum disorder assessment, using a mixed-method approach which involved both parents and staff completing a survey with closed and open-ended questions and/or parent one-on-one qualitative interviews or a focus group. The study highlighted the challenges faced by both clinical staff and parents during the assessment process. Despite the emotional challenges, parents felt supported; however, staff were self-critical about aspects of the process. It became clear that there is a need for a strong alliance between staff and parents throughout the process, which requires a holistic, adaptive, and collaborative approach. Staff challenges can be managed through a supportive multidisciplinary team, while provision of clear expectations prior to the assessment may alleviate parental stress.

## 1. Introduction

Autism Spectrum Disorders (ASD) are lifelong neurodevelopmental conditions, which, if undiagnosed or when diagnosis is delayed, can have a significant impact on outcomes for individuals and their families (Hedley et al., 2016). This can place pressure on clinical staff to provide a diagnosis to ensure early intervention is possible (Bono et al., 2021); however, for parents, the diagnostic process can be protracted and confusing, often resulting in elevated levels of parental anxiety (Makino et al., 2021; Yi et al., 2020). The individual challenges faced by both clinicians and parents are well reported; however, the similarities and differences between clinician and parent experiences have had little focus to date (Makino et al., 2021; Burden, 2024).

The Australian Bureau of Statistics (ABS, 2019) reported there were 205,200 people with an autism diagnosis in Australia, representing a 25.1% increase from 2015. Eighty-three per cent of those were under the age of 25 years, the highest prevalence being for those aged 5 to 14 years (Australian Institute of Health and Welfare, 2017). Given the young age of many children undergoing assessment for ASD, it is common for parents to attend the assessment process. It is therefore important that the parents are as well prepared as possible and given advance information about the process, especially the length of time needed for a thorough assessment, which may take up to two months for the various elements to be completed (Andersson et al., 2014).

### 1.1. Impact on Parents and Families

When parents have a positive experience of the diagnostic process, it can have a profound impact on their acceptance and understanding of the diagnosis (Bendik & Spicer-White, 2021). A positive experience can also have a beneficial impact on parents’ ability to cope with their child’s condition. However, there are many aspects which can negatively affect a parent’s satisfaction prior to and during the assessment phase. These include the length of time from expressing concern to obtaining a diagnosis, dissatisfaction with the information provided (Munro et al., 2023), feeling their child was labelled, rather than described, or when focus was placed on the child’s disabilities and symptoms, with no acknowledgement of their strengths and abilities (Bendik & Spicer-White, 2021). As a result, parental stress tends to be high, with feelings of loss, devastation, and mourning, regardless of how or from whom they received a diagnosis (Moodie-Dyer et al., 2014; Daly et al., 2026; MacKenzie et al., 2023). When parents feel overwhelmed or alienated by the assessment experience, they can often miss important information (Glick et al., 2024; Sommerfield et al., 2024). In contrast, parents report that they value services that are open and transparent in their communication, use non-medical language, value parents’ perspectives, collaborate effectively and show compassion, sensitivity and empathy towards both parent and child (Buzanko, 2018).

### 1.2. Impact on Clinicians

Despite there being a range of tools and clinical guidelines (Penner et al., 2018) available to help practitioners make an ASD diagnosis, it is often left to the expertise of the clinical team to interpret assessment results and formalise a diagnosis (Rogers et al., 2016). Clinician confidence is a key aspect of an accurate diagnosis; however, not all generalist mental health clinicians receive adequate training and support to assess and manage young people with autism (Hedley et al., 2016). In addition, training in family-focused care may be insufficient in undergraduate clinical training (Bailey et al., 2003). It is vital to understand specific gaps in knowledge and confidence levels to ensure provision of targeted education, training, professional development, and support (Bono et al., 2021; Porter et al., 2025). Where staff feel more confident, competent and supported, outcomes for children and families are likely to improve (Hedley et al., 2016; Howes et al., 2021).

### 1.3. Working Together

A scoping review by Makino et al. (2021) highlighted the experience of parents in the diagnostic process. They found a large number of studies described parents’ experiences of this process as uniquely stressful. However, their study did not indicate any evidence of the clinicians’ experiences in relation to those parents’ experiences. Despite this, it is known that the quality of support provided by clinicians and professionals in this space is critically important in reducing parental stress (Ilias et al., 2018; Legg & Tickle, 2019). To the authors’ knowledge, no study has examined parent views in conjunction with clinician views of the assessment process and what can be learnt or understood through each of these experiences. This study aims to begin addressing this gap in understanding of the ASD assessment process.

### 1.4. The Current Study

The tertiary community mental health service described in this study provides a specialised Developmental Assessment and Management Programme (DAMP) for complex children and young people with suspected neurodiversity and other comorbidities. Following referral, the programme provides:

- Intake;
- A tailored assessment process;
- Participation of parents via phone or video conferencing when not able to be physically present;
- Preparation of a full written report on the assessment process, including diagnosis where made;
- An opportunity for parents to read and comment on the draft multidisciplinary report;
- Ongoing treatment and support as needed post-diagnosis.

The aim of the current study was to examine and compare the views and experiences of both parents and staff on the developmental assessment process, and to shed light on the convergent and divergent experiences of each group.

The research questions guiding this project were:

1. What were the experiences of staff and parents who took part in the developmental assessment and management programme?
2. How did the experiences of parents and staff compare and contrast with each other?

## 2. Method

To address the aims of the project, this study utilised a mixed methods approach with a focus on qualitative data regarding participants’ experiences. A convergent data collection strategy was utilised (QUAL + quan) (Morse & Niehaus, 2009), where qualitative and quantitative data were collected first via an online survey, with versions for parents and staff, including both closed and open-ended questions. Subsequently, parents who completed the survey also had the additional option to participate in either a focus group or a one-on-one interview to further elucidate their qualitative responses. Participation in the focus group or interview was voluntary and self-selected via an opt-in process.

Data collection occurred in 2019, prior to the impact of the COVID-19 pandemic, which necessitated significant changes to the ways in which assessment services were delivered.

### 2.1. Participants

#### 2.1.1. Parents

Parents were eligible to participate if they had a child currently being assessed or whose child had been assessed in the previous three years at the clinic where the study took place. One hundred and nineteen parent/guardians were contacted via email and invited to participate by completing an anonymous online survey. To preserve anonymity, no further demographic data was collected beyond the respondent’s gender and relationship to the child. Parents had the option to tick a box and provide their contact details if they wished to participate in either an optional focus group or one-on-one interview. Consent for participation in the survey was implied by the parent clicking on the survey link provided. Twenty-five parents (20 mothers and 5 fathers) completed the quantitative questions in the survey relating to the referral and the assessment, while 24 parents (19 mothers and 5 fathers) completed the questions related to the report. Nineteen of the parents also completed at least one of the open-ended questions (17 mothers and 2 fathers). Of the 25, five also participated in the focus group and two completed a one-on-one interview. Written informed consent was obtained from all participants in the focus group and interviews, including consent to audio-record, transcribe and anonymise the group or interview, in line with the requirements of the health service’s Human Research Ethics Committee. All seven participants in the focus group or interview were female. Sixty per cent of the children being assessed were aged 5 to 8 years, 32% were aged 2 to 4 years, and the remainder were aged 9 years or over.

#### 2.1.2. Staff

All staff employed in the multidisciplinary DAMP team within the child and youth mental health service completed the survey (n = 9). Disciplines represented in the team included psychiatry, psychology, speech pathology, occupational therapy, nursing and social work, all with mental health qualifications. Six of the staff members had been with the team for at least two years, while the other three had been with the team for less than two years. All staff rated their views on the assessment process, including intake, assessment, and reporting. Staff also had the opportunity to provide comments on managing parental concerns and distress.

### 2.2. Procedure

#### 2.2.1. Online Survey

An online survey was created to capture the experiences of participants involved in the assessments, referrals, and reports associated with the DAMP process. This survey involved both open-ended qualitative questions and closed, scaled questions, where staff and parents could rate their level of agreement with a number of statements relating to different aspects of the DAMP process. As no validated measures were applicable to the specifics of this programme, the qualitative and quantitative survey items were created by clinical staff and were informed by clinical practice and knowledge of the DAMP processes. The specific items were developed to capture staff and parent views about the key aspects of DAMP processes. Both clinical staff and research team members reviewed the survey items for face validity and clinical relevance. Pilot testing with staff members was conducted to ensure clarity and ease of use.

#### 2.2.2. Interviews and Focus Groups

The interviews and focus groups followed a semi-structured format based around the stages of the DAMP, but still giving participants freedom to move beyond the confines of the questions (see Appendix A for focus group and interview questions).

### 2.3. Data Analysis

#### 2.3.1. Qualitative Analysis

All qualitative data captured from the interviews and focus groups were audio-recorded and transcribed verbatim using third-party transcribing software. All transcripts were checked for errors before being analysed. All qualitative data collected from the survey were compiled alongside the transcription data according to the different stages of the DAMP process: the referral, the assessment, and the report. All qualitative data were thematically analysed by the first two authors according to the six stages of thematic analysis (Braun & Clarke, 2006). This first entailed a reading and re-reading of the transcripts to become familiarised with the data (Stage 1), and further reading and initial independent coding of the data by the first two authors according to their potential relevance to the aims of the study (Stage 2). The preliminary codes were then grouped together into themes according to the stages of the DAMP and the extent of their levels of shared meaning (Stage 3). Themes were then reviewed by the whole research team for consistency, and any discrepancies were addressed based on their coherence and the extent to which they captured different but cogent constructs (Stage 4). These final themes were then named to capture their core meaning (Stage 5), and finally, text segments were used to illustrate the themes (Stage 6) (Braun & Clarke, 2006).

#### 2.3.2. Quantitative Analysis

To provide supporting evidence for the qualitative data, the quantitative survey data were analysed using SPSS (Version 28) statistical software to assess the frequency of responses. Following this, a series of Mann–Whitney U tests were conducted to determine whether there were any differences between the staff and parent responses to the quantitative items. The decision was made to use Mann–Whitney tests as the responses to these items were ordinal responses to a Likert scale, and the responses were not expected to be normally distributed; thus, the nonparametric U test was chosen. Assumptions for the Mann–Whitney test were mostly met, with all data being ordinal, the groups being independent, and there being independence of observations. To test the assumption of approximately similar distributions, box plots centred on the median scores for each item were viewed. Most items were approximately similarly distributed except “the assessment wasn’t what I was expecting” and “I was given sufficient notice for the appointments” in the assessment section, and the items “The written report provided information I needed,” “the report was too long,” and “I had enough time to read and understand the report before my final meeting with staff” were slightly differently distributed. Thus, results relating to these items must be interpreted as comparing the overall distributions, rather than comparing the group medians. To measure the significance and strength of the results, the *p* value (exact) was chosen due to the small sample size and the rank biserial correlation coefficient was chosen as a measure of effect size (Fiel Peres, 2026).

#### 2.3.3. Integration of Qualitative and Quantitative Components

Both the qualitative and quantitative components of this study were designed to elicit the experiences of parents and staff regarding taking part in an ASD assessment. Thus, the two data sets complement each other, where the qualitative findings draw out the important aspects of the assessments for parents, while the quantitative findings offer both converging and diverging views of taking part (O’Cathain, 2020). Following the qualitative thematic analysis and quantitative analysis processes, three of the authors (MK, LH, RW) came together to triangulate the two data sets.

## 3. Results

The results of this study are presented in the order in which participants experienced them during the assessment process, namely: referral process; assessment process; reporting process. Qualitative themes are introduced first in each section with supporting quantitative data, then described.

### 3.1. The Referral

There were three main themes identified in the data relating to the referral process: length of time; finding the right person; and confidence in the team.

#### 3.1.1. Time

When parents spoke of the time from referral to assessment, there was a discrepancy, with some feeling the referral process was slow, “the actual referral for us was about six months”, and others saying, “the time between we sent the referral and the team contacted us was surprisingly fast”. Many of the parents indicated a level of frustration around this process, including “a few false starts”.

#### 3.1.2. Finding the ‘Right Person’

Many parents described the need to find the right team, and the right place to have their child assessed. One parent said, “[we] were looking for a multi-disciplinary therapist … we didn’t want our daughter to fall through the gaps” and another identified that after a prior misdiagnosis, “I was really quite stressed about the process and finding the right person”. These comments highlight the importance of the therapeutic alliance in achieving good outcomes in this kind of work.

#### 3.1.3. Confidence

Once the referral process had commenced, many parents described feelings of positivity around the intake, which instilled a level of confidence in them. For example:

“They were fantastic, I’ve never met such a professional team before. They were great, any question we had they answered …, they’ll call me and speak about it or our next appointment what it’s going to involve…”

To provide a quantitative picture of parents’ views about the referral process, Table 1 shows the frequency of parent responses when asked to reflect on their experiences. Nearly all agreed or strongly agreed that they were happy with the referral process, although 48% indicated they were anxious about what would be involved. Ninety-two per cent felt they were able to ask questions before the assessment, and 96% felt that staff listened and understood their concerns. Note that treating staff were not asked about the referral process.

#### 3.1.4. Integration of the Referral Findings

Comparison of the themes found in the qualitative data regarding the referral process with the responses to survey items revealed a high degree of convergence (see Table 2).

### 3.2. The Assessment

There were three themes identified by both parents and staff in relation to experiences of the assessment: the challenges; the duration of the assessment; and preparedness.

#### 3.2.1. Challenges

When parents spoke of the challenges, there were two primary aspects: the emotion of their child and their own concerns. Many parents felt that the assessment process had a deep impact on their child, “ours was traumatic from beginning to end. It took three months … the whole thing. My son was in deep trauma for the entire time”. Another parent described how the process left their child reluctant to attend, “He never wanted to come into the building. He knew he was going to be poked and prodded, but we were upfront with him”. Parental comments reflected their concerns that the emotion of their child may have an effect on the assessment process and may not be an accurate reflection of their child: “We kept worrying that it wouldn’t be a reasonable assessment of what he’s like…”.

For another, the challenge was managing the child’s day following their assessment session: “He’s absolutely totally wiped out. He’s exhausted. He’s had enough”. Parents also expressed that they found it “emotionally more challenging… because of the fact that by its nature you’re focusing on the challenges your child has”.

Staff also identified challenges in regard to the assessment such as the time needed to coordinate multiple appointments “when the team is part-time, and parents are working” and supporting families through the process “responding sensitively to the parent’s needs at the same time as managing a child with dysregulated behaviour, poor attention or low self-esteem”.

#### 3.2.2. Duration

The parents who participated were divided on the duration of the assessment, with some feeling “The length of time was good” and others feeling “I definitely felt as though the assessment was way too long”. This discrepancy may reflect a lack of communication between the treating team and the family about the time commitment required to complete the assessment; however, it may also reflect personal differences and preferences between individuals and families, reflecting the need for person-centred care to accommodate individual tolerances where possible. Staff also identified some structural issues in timing, whereby some cases were prolonged due to “coordinating multiple appointment[s]”.

#### 3.2.3. Feeling Prepared

Parents felt there could be some improvement in the way both they and their child were prepared for the assessment. There was some level of uncertainty around what to expect, “knowing what to expect and what tasks will be involved helps her in being comfortable with new situations and requirements” and how to prepare for the assessment, “You try to prepare them for it, but you can’t. Cos it’s not like anything they’ve done before. The reason you are there is because everything has gone to shit anyway”.

Despite some of the difficulties, other parents acknowledged the support they received and the importance of the emotional support from staff. One parent commented, “I was nervous as a mum. I didn’t know how hard it was going to be. But they were fantastic. They were caring and they were comforting me saying “it’s going to be alright”.

Quantitative findings from the survey regarding the assessment provided some support to the qualitative findings. Both parents and staff responded to nine statements about the assessment and rated their level of agreement or disagreement with each one. Results of the Mann–Whitney U tests indicated both convergence and divergence in responses between parents and staff (see Table 3). Although there was no difference in views between parents and staff in relation to the assessment process, regarding things such as feeling understood and respected, there was a statistically significant difference between staff and parents’ views about how much notice was given for the assessments, with staff feeling that the notice given was less sufficient than parents.

#### 3.2.4. Integration of the Assessment Findings

Comparion of the qualitative data regarding the assessment process with the survey responses also revealed a high degree of convergence in responses, although the qualitative data regarding the feeling of being underpreparaed for the process helps to explain low agreement with this item (see Table 4).

### 3.3. The Report

Analysis of the qualitative data regarding the process of providing and receiving the report of the assessment found four main themes: language used in the report; the length and purpose of the report; the content of the report; actions going forward.

#### 3.3.1. Language

Many of the parents felt the language used in the report was “highly clinical”, which they found “confronting”. One parent commented, “There were some (aspects) written in highly clinical language … they used the word disability quite a bit. We didn’t know autism was classified as a disability”. Another noted that “there were too many big words”.

In contrast, the staff felt that they “try and use plain English” but a few identified this as an area that “we can do better”, acknowledging that the report was “not easy for a lay person to understand”. Despite this, the staff felt the formality of the language used in the report was necessary due to the variety of audiences such as the family, school and other therapists.

#### 3.3.2. Length and Purpose

Although some parents found the report “a bit too long”, others felt it was “comprehensive”, acknowledging the necessity for the level of detail. However, parents recommended that it would be beneficial to have a shorter 2-page version for their child’s school. One parent identified that they had received the report in different versions for teachers and medical professionals, which they were happy with.

Many of the staff agreed with the parents and felt the report was too long; however, one person felt this was necessary since it was “multidisciplinary”. On the other hand, another staff member felt that the length of the report made their “workload unrealistic”, especially since they felt that “a large number of parents and professional don’t read large parts of the report”.

#### 3.3.3. Content

One parent suggested that the report needs to be more “strength focused” with “recommendations” as opposed to it being “negative in the way it was presented”. Many of the staff acknowledged the difficulties parents faced “to read about their child’s problems in the report”. Some staff also felt the report was not “strength-based”, and this was due to the purpose of the report, which meant writing about strengths was “unrealistic”.

#### 3.3.4. Going Forward

Many of the parents felt there was not enough information for them following the assessment: “I wasn’t clear about that going forward. That was probably the only aspect of those recommendations that was a bit confusing perhaps at the time”. Another felt the recommendations at the end were “a bit vague”. In contrast, another parent felt “relieved” and said the staff were “very helpful, they gave us instructions on what to do next and they were very supportive in that as well”.

Staff also reported that some parents wanted “more detail in the recommendations about what to do specifically” whereas the reports are usually written with “broad suggestions”.

Overall, staff felt that “working in a multi-disciplinary team with clear roles and great teamwork” provided them with the support they needed in their work.

In the quantitative section of the survey relating to the report, both parents and staff responded to nine statements regarding the written report provided to parents following the assessment process. As with the assessment phase, there was both convergence and divergence between staff and parents’ responses (see Table 5). Staff and parents mostly agreed in relation to the report accurately describing the child, having enough time to read the report, having useful recommendations, feeling supported by staff, focusing on the strengths of the child, and giving hope to parents. However, there was a significant difference in agreement regarding the extent to which the report was clear and easy to understand, was the appropriate length, and included appropriate information needed by parents. Overall, the data indicated that staff were generally more critical of the process compared to parents.

#### 3.3.5. Integration of the Report Findings

Examination of the qualitative themes and the relevant quantitive items regarding the report revealed the lowest level of agreement between parents and staff, when compared to the other aspects of the process (see Table 6).

## 4. Discussion

The findings of the current study highlight the emotional challenges for children, parents and staff involved in providing timely, useful, and practical ASD assessments. While the response rate for parents to the survey was low (21%), it is not uncommon for surveys of this type, especially when inviting parents who were caring for a child with challenging behaviours (whether they received a diagnosis or not), and was regarding an experience that was likely stressful for parents to undertake. While this may introduce a degree of bias in the respondents, we cannot be sure of the direction of selection bias, whether towards parents who were most negatively or most positively affected. The range of responses in the data suggests that a wide range of experiences of care was captured in the study.

Parents in this study reported the emotional impact they and their child experienced, even when feeling supported by the team through the process (Rabbitte et al., 2017; Legg & Tickle, 2019). The data indicate that while parents and staff agreed on many aspects of the process, there was disagreement on views such as the amount of notice provided for assessments, and the length and content of the written report. Staff and parents rated the preparation for the assessment as an area which could be improved. Qualitative responses indicated that the written report on the child was not sufficiently strengths-based and didn’t provide hope.

Other issues which were identified included the need to balance professional and clinical information with user-friendly language or alternative documentation for different audiences, as well as scope to ensure a balance is achieved between the child’s strengths and deficits identified through the assessment process (Makino et al., 2021). This aspect is critical since parents who report a more positive experience through the assessment process are more likely to cope with and accept any diagnosis given (Siklos & Kerns, 2007), thereby reducing parental stress levels and improving outcomes for both child and parent (Buzanko, 2018). Interestingly, staff appeared to be more critical of the process, perhaps due to their recognition of the importance of the parental experience and their responsibility to make this as positive as possible.

Assessing and diagnosing ASD in children can be equally challenging for staff due to the complex nature of the work and skill set needed (Yates & Le Couteur, 2016; Howes et al., 2021). Although experience is a key aspect of managing these complexities, the level of staff confidence can increase the accuracy of the diagnosis (Hedley et al., 2016). Clinicians in this study indicated that they felt supported by working as part of a multi-disciplinary team, which has been suggested elsewhere to be a means of improving the accuracy of the assessment (Hedley et al., 2016).

Additional challenges for staff in the current study included managing the intense emotional nature of the work, family expectations, and, in particular, parental stress. This can be challenging when staff are unable to ascertain whether parental stress and dissatisfaction are related to the process of diagnosis or the diagnosis itself (Braiden et al., 2010; McCrimmon & Gray, 2021).

A further issue identified by parents was the stress caused by bringing their child into the clinic, where the child’s behaviour may be different from what the parent has observed at home or school. While it may seem useful to recommend that more outreach services are available to observe and assess children in their everyday environments (Ng et al., 2022), the service in this study was not funded to provide outreach assessments at the time of the study. The shift to telehealth following the COVID-19 pandemic may go some way to alleviating this issue in the future, whilst also recognising the additional challenges introduced by the telehealth modality (Harrison et al., 2025).

## 5. Limitations

It is important to acknowledge some limitations with the quantitative aspects of the survey. The survey tool was designed specifically for this study and has not been externally validated. The questions asked to parents and staff were not identical, which may have impacted results; however, these questions were designed with the input of staff to best capture their opinions on the important aspects of the assessments and reports. Further, the sample sizes in the present study were small, unevenly distributed between respondent groups (parents and staff) and limited to one clinic, which may have impacted the significance and generalizability of the results, and thus must be interpreted with some caution. However, the real-world constraints of researching programmes such as this meant that sample sizes were capped at the number of clients and staff members involved in the program. Despite these limitations, we feel the quantitative component of the study adds support to the more nuanced qualitative findings.

The response rate for parents was also low, and parent experiences varied across the cohort; as such, results should be interpreted with caution. In addition, many of the participants were mothers. Parental stress can manifest differently between mothers and fathers (Ilias et al., 2018), so future research should consider further examination of the experience of fathers. In addition, the study was also reliant on parental recollection of events, which for some was up to three years prior. It is possible that only the aspects of the assessment that made a direct emotional impact on parents or families were reported. The response rate therefore may be indicative of the stress that parents described in the study, resulting in a reluctance to revisit this stressful period in time. An implication for services undertaking neurodevelopmental assessment might be consideration for the assessment to occur as outreach in a place more familiar to the child and parent/carer to alleviate some of the reported stress. However, this may also hold implications for service delivery due to the need for multidisciplinary staffing and suitability of the environment for observation.

Some of the staff at the time were new to the team and the process involved, so this was only indicative of their experiences at the time. Repeating the data collection with a more experienced workforce within the team may yield different results. The voice of the child or young person was also not captured, and involvement of their experiences through the process would be informative and add an additional layer of understanding for service providers.

## 6. Conclusions

Evidence shows that undertaking an ASD assessment can be challenging for children, parents, and clinicians alike. It requires staff to be confident in their clinical skills and to feel emotionally supported by their team and service management. Parents’ perceptions of being supported are predictive of more positive parental coping strategies (Liu et al., 2023), which are in turn more likely to lead to improved confidence and better adherence to follow-up interventions (Cai et al., 2025). The current study highlights the importance of recognising differences in experiences between the clinician or professional staff and parents to ensure a cohesive approach to developmental assessment and diagnosis. When staff feel supported and have confidence in their work, they are more likely to be able to support parents through their distress (Ilias et al., 2018). This study has helped to demonstrate areas where clinicians may have a different experience or understanding of the assessment process and the ways in which the assessment is experienced by parents who go through it. Using shared language, taking a strengths-based approach, offering person-centred care and managing the time burden for parents are all factors which services can implement to improve families’ experiences. In addition, more work in the preparatory space may be helpful in managing parental expectations as well as the demands on the child.

## Figures and Tables

**Table 1:** Parent views of the referral process.

	Strongly Disagree	Disagree	Neither Agree nor Disagree	Agree	Strongly Agree	Not Sure	No Response
I was happy to be referred for assessment at this service	0%	0%	4%	36%	60%	0%	0%
I was anxious about what the assessment would involve	4%	36%	12%	36%	12%	0%	0%
I was anxious about receiving a diagnosis for my child	4%	28%	0%	32%	32%	0%	4%
I thought that receiving a diagnosis would be helpful for my child	4%	4%	8%	36%	44%	0%	4%
The information which I got before the assessment took place was clear and easy to understand	0%	0%	8%	40%	48%	0%	4%
I was able to ask questions before the assessment took place	0%	4%	0%	56%	36%	4%	0%
Staff listened to me and understood my concerns	0%	0%	0%	36%	60%	4%	0%

**Table 2:** Integration of qualitative themes and quantitative items.

Qualitative Theme	Relevant Quantitative Item	Complementarity/Convergence/Divergence
Time	Not asked directly	
Finding the right person	Staff listened to me and understood my concerns	For parents, connecting with the service and trusting the treating team were important aspects of the assessment process.
Confidence	The information which I got before the assessment took place was clear and easy to understand	Both qualitative and quantitative data highlighted the importance of parents feeling heard and having confidence in the service

**Table 3:** Mann–Whitney *U* tests comparing staff and parent views of the assessment.

Parent/Staff Survey Question	Parents (N = 25)		Staff (N = 9)		z	*p* (Exact)	Mann–Whitney U	r_rb_
	M	Range	M	Range				
	(SD)		(SD)					
Staff understood my concerns/I felt I understood the parents’ concerns	3.36 (0.70)	(2–4)	3.44 (0.53)	(3–4)	−0.152	0.908	109.0	−0.03
Staff respected my opinions/I felt I respected the parents’ opinions	3.44 (0.58)	(2–4)	3.56 (0.53)	(3–4)	−0.467	0.701	102.0	−0.09
Staff were sensitive to my needs/I felt I was sensitive to the parents’ needs	3.28 (0.74)	(2–4)	3.44 (0.73)	(2–4)	−0.597	0.591	98.5	−0.12
The staff didn’t respond appropriately to my child/I always responded to the child appropriately *	3.08 (1.04)	(0–4)	3.22 (0.67)	(2–4)	−0.042	0.969	111.5	−0.01
I didn’t feel properly prepared for the assessment/I was always prepared for the assessment *	2.96 (0.89)	(0–4)	2.89 (0.60)	(2–4)	−0.470	0.673	101.5	0.10
The assessment wasn’t what I was expecting/I ensured the parent knew what to expect *	2.76 (1.13)	(0–4)	2.78 (0.44)	(2–3)	−0.206	0.848	107.5	−0.04
I was given sufficient notice for the appointments/I always ensured the parent was given sufficient notice for appointments	3.40 (0.65)	(2–4)	2.56 (0.88)	(1–4)	−2.553	0.017	52.0	0.54
The number of weeks to complete the assessment was too long/I think the number of weeks for the assessments was about right *	2.72 (1.06)	(0–4)	2.22 (0.83)	(1–3)	−1.415	0.188	78.0	0.31
The assessment gave me a better understanding of my child and how to support him/her/I aimed to give the parent a better understanding of their child	3.44 (0.71)	(2–4)	3.22 (0.68)	(2–4)	−0.949	0.397	90.5	0.20

* Items were reverse-coded.

**Table 4:** Integration of the qualitative themes and quantitative items.

Qualitative Theme	Relevant Quantitative Item	Complementarity/Convergence/Divergence
Challenges	The staff didn’t respond appropriately to my child/I always responded to the child appropriately	Parents felt that there were challenges for both themselves and their child with the process, particularly emotional challenges, but that staff responded appropriately to their child.
Duration	The number of weeks to complete the assessment was too long/I think the number of weeks for the assessments was about right	On average, both parents and staff felt that the assessment process was long, but not excessively long; however, this item had one of the lowest rates of agreement among all participants. Qualitatively, some parents definitely felt that it had taken too long.
Feeling prepared	I didn’t feel properly prepared for the assessment/I was always prepared for the assessment	Feeling properly prepared was one of the lower-rated items in the survey, both for parents and staff, indicating both felt there was room for improvement in the preparation provided. Despite this, qualitative feedback suggests that this may be an inevitable part of such an intensive assessment which hasn’t been undertaken before.

**Table 5:** Mann–Whitney U test comparing staff and parent views of the written report.

Parent/Staff Question	Parents (N = 24)		Staff (N = 9)		z	*p* (Exact)	Mann–Whitney U	r_rb_
	M	Range	M	Range				
	(SD)		(SD)					
The written report provided the information I needed/I think the reports provided parents with the information they need	3.42 (0.72)	(1–4)	2.78 (0.44)	(2–3)	−2.75	0.012	47.5	0.56
The written report was too long/I think the reports were too long	1.17 (0.82)	(0–3)	2.44 (1.13)	(1–4)	−2.91	0.005	40.0	−0.63
I had enough time to read and understand the report before my final meeting with staff/I think the parents had enough time to read and understand the report before their final meeting	3.33 (0.76)	(1–4)	2.78 (0.67)	(2–4)	−2.01	0.054	60.5	0.44
I think that the written report described my child accurately/I think the reports accurately represented the child	3.08 (0.93)	(0–4)	3.33 (0.71)	(2–4)	−0.641	0.564	93.5	−0.13
The recommendations in the report were useful to me/I aimed to give useful recommendations in the report	3.13 (0.74)	(1–4)	3.00 (1.00)	(1–4)	−0.180	0.890	104.0	0.04
I didn’t feel supported and understood by the staff during the first feedback meeting/I aimed to support and understand the parents in the feedback session *	3.04 (1.04)	(0–4)	3.33 (0.71)	(2–4)	−0.588	0.592	94.5	−0.13
I think the feedback focused on strengths/I think the reports focused on the strengths of the child	2.58 (0.93)	(1–4)	2.22 (0.97)	(1–3)	−0.811	0.462	89.0	0.18
The report gave me hope/I think the report gave hope to the families	2.54 (1.02)	(1–4)	2.11 (0.78)	(1–3)	−1.08	0.309	82.5	0.24
The written report was clear and easy to understand/I think our reports are clear and easy to read	3.54 (0.59)	(2–4)	2.56 (0.73)	(1–3)	−3.28	0.002	34	0.69

* Items were reverse-coded.

**Table 6:** Integration of the qualitative themes and quantitative items.

Qualitative Theme	Relevant Quantitative Item	Complementarity/Convergence/Divergence
Language	The written report was clear and easy to understand/I think our reports are clear and easy to read	This was one of the areas in which there was disagreement between parents and staff. In addition, some parents found the language confronting, while staff felt that the language was appropriate for the variety of contexts in which it would be used.
Length and purpose	The written report was too long/I think the reports were too long	This was another area in which there was disagreement between parents and staff, and between some parents who thought the report was too long and some who thought it was important to be comprehensive.
Content	The written report provided the information I needed/I think the reports provided parents with the information they need	Quantitatively, both parents and staff agreed that the report content was useful; however, the qualitative findings unpacked the impact of deficit-focussed content in the report.
Going forward	The report gave me hope/I think the report gave hope to the families	Parents rated this item one of the lowest on the survey, and unpacked this further in the qualitative findings through feeling they were unprepared for what was coming next.

## Data Availability

The original contributions presented in this study are included in the article. Further inquiries can be directed to the corresponding author.

## Appendix A. Developmental Assessment Focus Group/Interview Questions

**Section 1: Demographic information**

1. Your role
  - Mother
  - Father
  - Grandparent
  - Guardian
  - Other (please specify)
2. How old was your child at the time of their DAMP assessment (please tell us about the latest assessment if your child has been assessed more than once)?
  - 2–4
  - 5–8
  - 9–12
  - 13+
3. Is your child
  - Boy
  - Girl

**Section 2: Intake and referral**
  First we would like you to think about your initial contact with the developmental assessment team and the referral.

1. Please can you tell me about your experience of being referred to the team
  - How did you feel about the referral and the process?
  - How clear was the information you were provided with?
  - Do you feel the staff listened to you and your concerns? Can you give an exam-ple?
  Prompt:
  Were you offered the opportunity to ask questions?
  Was the information provided too little or too much? Why?

**Section 3: About the assessment**
  Now think a back to when you your child was being assessed.

1. What was your experience of the assessment process?
  Prompt:
  How was any distress managed?
  Do you feel listened to?
2. How well prepared did you feel about the assessment?
  Prompt:
  Did you have enough information?
  Was your child prepared?
3. How do you feel about the length of time for the sessions?
  Prompt:
  e.g., too long/ too short/ rushed
4. If you could have changed anything about the assessment process what would it be?
  Prompt:
  Would you have liked the option for sessions at home or elsewhere?
5. What was your experience of the staff who conducted the assessment?
  Prompt:
  Was there consistency in the team and did you know who they were?

**Section 4: About the information and report**
  Thinking about the information you received and the report at the completion of your child’s assessment.

1. What was your experience of receiving the report?
  Prompt:
  e.g., useful, clear, easy to understand, too short, too long etc
2. What was your experience of the two face-to-face meetings?
3. How did you find the recommendations provided?
  Prompt:
  Did you know what to do with the report/recommendations?

**Section 5: Overall satisfaction**

1. If you could change anything what would it be?
2. Overall, how satisfied were you with the experience?
3. Is there anything else you would like to tell me?

**Thank you for participating**
